# Spontaneous lumbar intraspinal subdural abscess: a case report

**DOI:** 10.1186/s13256-023-03872-7

**Published:** 2023-04-01

**Authors:** Oscar James MacCormac, Nabih Berjaoui, Sean Mizzi, Difei Wang, Sabina Patel, Qusai Al Banna, Cristina Bleil

**Affiliations:** 1 School of Biomedical Engineering and Imaging Sciences, King’s College London, St Thomas’ Hospital, London, SE1 7EH UK; 2 Department of Neurosurgery, King’s College Hospital, Denmark Hill, London, SE5 9RS UK; 3 Department of Cardiothoracic Surgery, St Thomas’ Hospital, Lambeth Palace Road, London, SE1 7EH UK

**Keywords:** Subdural, Empyema, Infection, Abscess, Case report

## Abstract

**Background:**

Subdural spinous abscess is a rare pathology that carries significant morbidity if not diagnosed and treated early; of the cases reported in the literature, very few are genuinely spontaneous in nature.

**Case presentation:**

Here we demonstrate the case of an otherwise entirely fit and well 56-year-old White, British female presenting with low back pain, bilateral sciatica and sensate urinary retention; lumbar subdural spinous abscess was diagnosed on urgent magnetic resonance imaging and the patient was successfully managed with surgical evacuation and prolonged antibiotic therapy. The patient made a full neurological recovery and was followed-up in the outpatient setting 12 weeks following her initial surgery; she was pain free with normal inflammatory markers and a normal neurological examination. There have been no further consultations and a telephone call at 20 weeks confirmed that she remains well.

**Conclusions:**

This is the second case reported in the literature of a genuinely spontaneous subdural spinous abscess, which was successfully managed with surgical evacuation following prompt diagnosis. This highlights the need to ensure infective pathologies are kept at the back of one’s mind even in the most unlikely circumstances, and that excellent outcomes can be achieved with early surgical intervention.

## Background and importance

Spinal epidural abscess (SEA) is a well-recognized clinical pathology, often associated with hematogenous spread of infection from sources outside of the spine [1]; however, spinal subdural abscess (SSA) is far rarer [2] and potentially more difficult to diagnose.

There are approximately 80 reported cases of SSA in the literature [2–5], which means the incidence of this rare condition is not yet known. The majority of cases seem to be associated with a secondary cause[2], such as hematogenous spread of infection [6–9], spread from decubitus ulcers (pressure sores) [10], epidermoid cysts [11], dermal sinus tracts [12], and iatrogenic (such as postoperative, following insertion of spinal catheters and even acupuncture) [13–15]. Cases that are not associated with any of the above, and thus present with a primary SSA, are incredibly uncommon, with only one previous case reported in the lumbar–sacral region to date [8].

Although rare, the consequences associated with untreated SSAs are significant, including neurological deficit [16] and death [10]; therefore, early recognition and treatment (often involving surgical drainage and prolonged antibiotic therapy [5]) is essential to achieving good patient outcomes.

Here we present our own case of a successfully managed spontaneous SSA in an otherwise fit and well female in her sixth decade of life.

## Case presentation

A 56-year-old White, British female was referred from her local hospital to our regional neurosurgical center with a 4-week history of progressive lower back pain and sciatica, associated with (in the latter 2 weeks) lower limb weakness and sensate urinary retention and incontinence, as well as fatigue and general malaise. An unenhanced magnetic resonance imaging (MRI) of the lumbar–sacral spine was performed, demonstrating an intradural T2-hyperintense lesion displacing the cauda equina at the L4–S1 vertebral levels.

The patient was afebrile on presentation; however, blood tests revealed a white cell count (WCC) of 24.42 × 10^9^/L, with a neutrophilia of 19.66 × 10^9^/L. MRI also demonstrated an enlarged bladder and so the patient was catheterized, draining 675 ml; she was able to feel the catheter pass. She had no medical history of note and was, until this point, very active, fit, and well.

The patient was subsequently transferred to our regional neurosurgical center for assessment and overnight contrast-enhanced imaging. On arrival she was assessed by the neurosurgical team and found to have the following:

**Table:** 

Myotome	MRC grade left	MRC grade right
L1/2	4	4
L3	4	4
L4/5	4+	4+
L5	3	4
S1	3	3

Reflexes were brisk in knee extension and ankle jerk bilaterally, plantars remained downgoing, and sensation was reduced to light touch in the L5 and S1 dermatomes bilaterally. Perianal sensation was intact to both light touch and pin prick, and anal tone was found to be normal on digital rectal examination. The remainder of the neurological examination was normal.

**Fig. 1:**
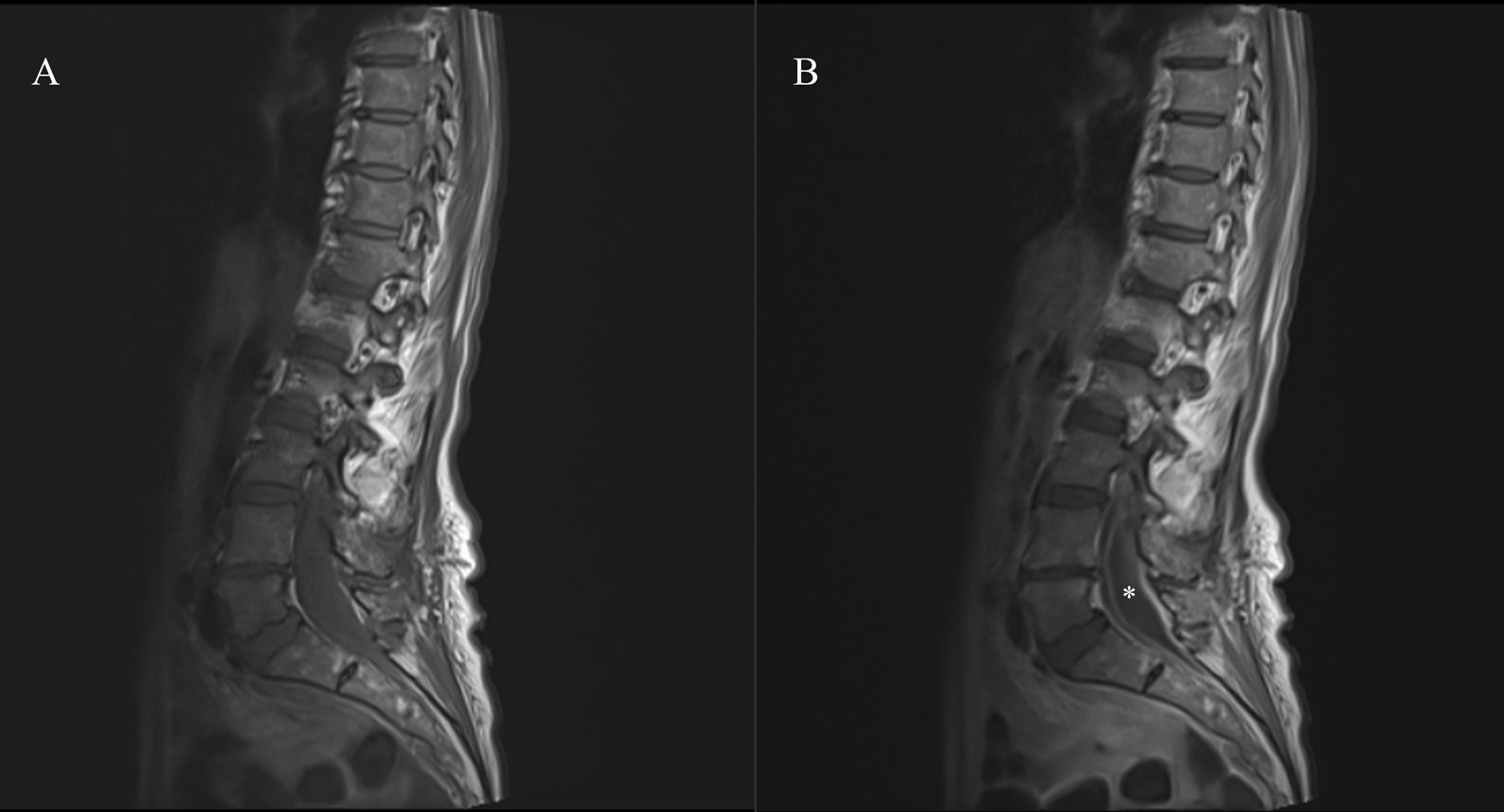
**A** Preoperative unenhanced sagittal T1-weighted MRI of the lumbar–sacral spine. **B** Contrast-enhanced sagittal T1-weighted MRI of the lumbar–sacral spine demonstrating enhancing collection (*)

Contrast enhanced MRI imaging of the whole spine was performed out of hours, showing that the previously demonstrated collection was peripherally enhancing (Fig. 1), thus indicating a potentially infective process (although a ruptured spinal dermoid/epidermoid cyst remained in the differentials). The patient was commenced on intravenous broad-spectrum antibiotics with central nervous system (CNS) penetration [intravenous ceftriaxone 2 g once daily and intravenous vancomycin 1.5 g (adjusted according to level checks) once daily] at this point.

**Fig. 2:**
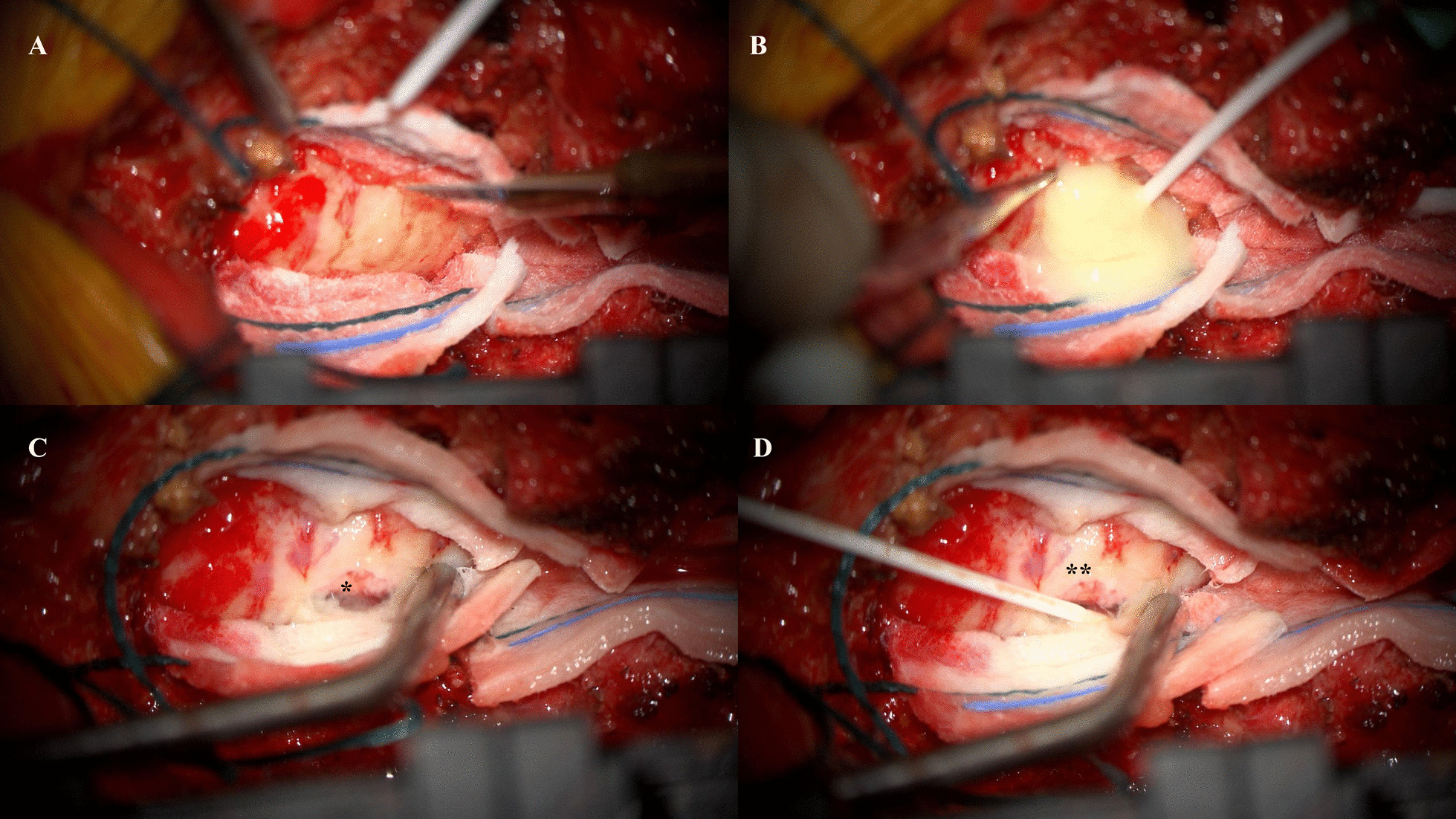
**A** Thickened, inflamed dura mater **B** Intradural pus following durotomy **C** Hyperemic nerve root (*), **D** Organized, adherent pus not removed (**)

Given the patient’s good premorbid health status and evidence of neurological compromise, a decision was made to surgically evacuate the presumed subdural abscess. The patient was taken to the operating theater and right-sided hemilaminectomies of L4, L5, and the top half of S1 were performed to gain access. The underlying dura was noted to be thickened and inflamed (Fig. 2A) and, following a small durotomy at the L5/S1 level, a large volume of frank pus was released from the subdural space (Fig. 2B)

The subdural space was washed thoroughly with saline and a severely hyperemic nerve root was visualized (Fig. 2C) and protected. Organized and very adherent pus (Fig. 2D) was left *in situ* due to the risk of neural injury from too aggressive a removal. The dura was closed with 5-0 polydioxanone suture (PDS) and overlain with a hemostatic patch (Hemopatch).

The patient was continued on intravenous ceftriaxone and vancomycin and has made an excellent neurological recovery, with no pain, deficits, or postoperative complications. Initial cultures were negative for a causative organism, however 16S ribosomal DNA reverse transcriptase polymerase chain reaction (rDNA RT–PCR) revealed a 99% sequence homology to *Streptococcus* species *S. pneumoniae*, *S. mitis*, *S. pseudopneumoniae*, and *S. oralis*.

**Fig. 3:**
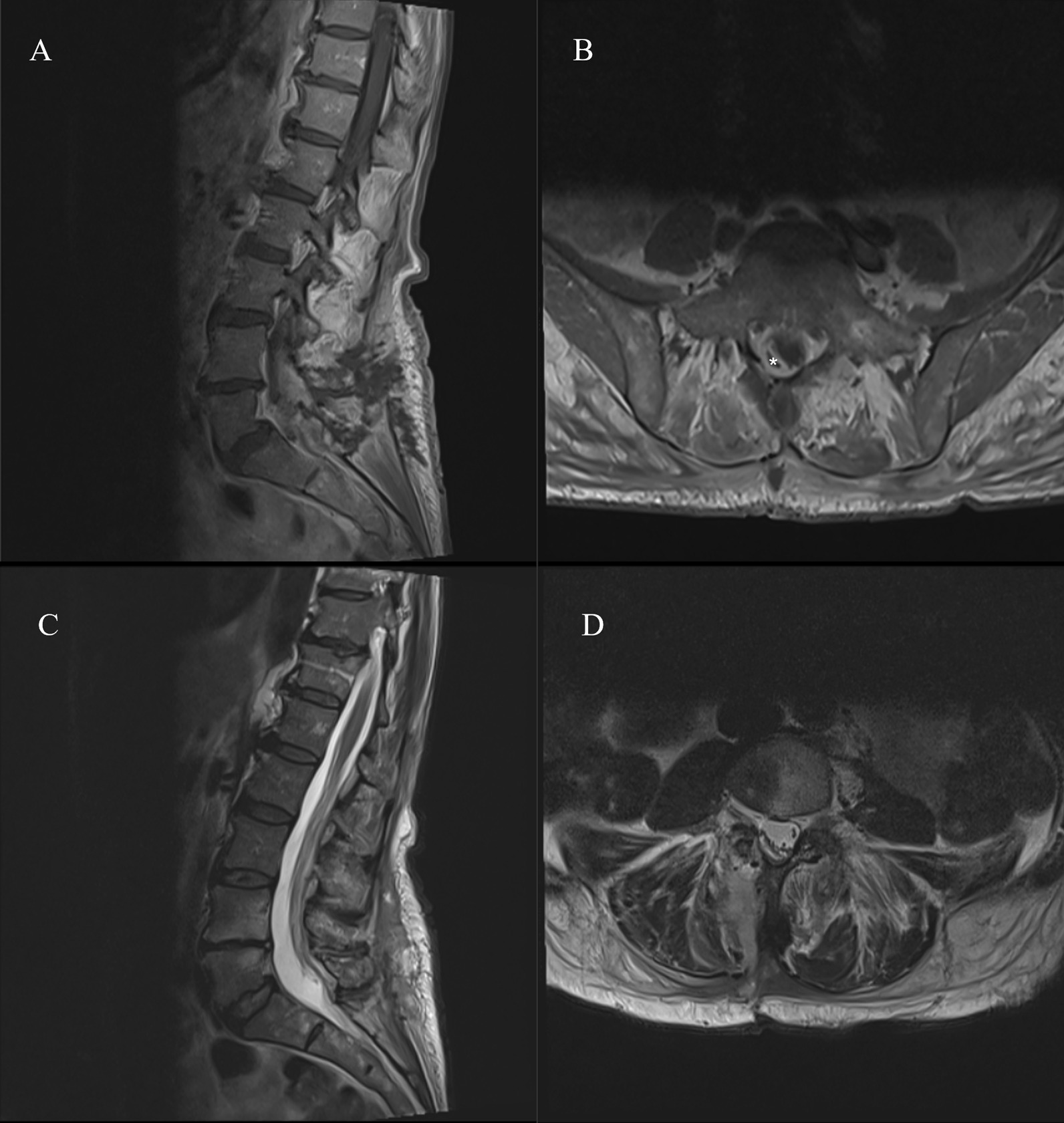
**A** Postoperative contrast-enhanced sagittal T1-weighted MRI of the lumbar-sacral spine **B** Postoperative contrast-enhanced axial T1-weighted MRI at the L4/5 level demonstrating a small residual enhancing collection (*) **C** Postoperative midline sagittal T2-weighted MRI of the lumbar-sacral spine demonstrating marked reduction in size of the preoperative collection **D** Postoperative axial T2-weighted MRI of the L4/5 level demonstrating marked reduction in size of the preoperative collection as well as small residual

Repeat MRI imaging performed 2 weeks postoperatively demonstrated excellent evacuation of the abscess (Fig. 3) with a very small residual collection that was managed with long-term (8 weeks) intravenous antibiotics (ceftriaxone 1 g once daily), delivered in an outpatient setting. The patient made a full neurological recovery and was followed-up in the outpatient setting 12 weeks following her initial surgery; she was pain free with normal inflammatory markers and a normal neurological examination. There have been no further consultations and a telephone call at 20 weeks confirmed that she remains well.

## Discussion

The rarity of this case highlights a challenging diagnosis, with potentially very severe consequences, as demonstrated in the literature [10, 16]. It is generally accepted among surgeons that the optimal treatment for abscess is drainage, given the challenges associated with antibiotic penetrance; however, as discussed in the background information, the reported cases are so often associated with secondary causes in patients with significant comorbidities that surgery is a very high risk or even nonviable option [2]. It is therefore crucial, where possible, to demonstrate that this pathology can and should be treated with prompt surgical decompression and abscess drainage in patients where this is safe, and it should be the first line regimen in these cases.

## Conclusions

This case demonstrates an excellent outcome in a patient with an extremely rare yet dangerous spontaneous lumbar spinal subdural abscess, highlighting the paramount importance of early diagnosis and prompt intervention to prevent progression, neurological deterioration, and even death. It is essential for all clinicians to keep an infective process in mind when considering the holistic clinical picture, especially when the initial spinal imaging is not clear and certainly not as expected; gadolinium contrast-enhanced MRI imaging remains the gold standard in aiding diagnosis for these cases and may not routinely be performed unless infection is specifically stated as a potential diagnosis.

This case also demonstrates the clear role for surgical evacuation in patients with a good premorbid health status. In cases where the SSA is demonstrated to be secondary to another cause, which may preclude patient suitability for surgical intervention [2], this can be a challenging decision-making process; however, in this extremely rare case where the SSA was deemed to be genuinely spontaneous, the role for prompt surgical evacuation is evident.

## Data Availability

Not applicable.
